# Supplementary material for: Flow-induced periodic chiral structures in an achiral nematic liquid crystal
Source: Nat Commun. 2024 Jan 8;15:7. doi: 10.1038/s41467-023-43978-6 (PMC10774319; doi:10.1038/s41467-023-43978-6)
Supplement: Supplementary file 1 — Supplementary Information [file 41467_2023_43978_MOESM1_ESM.pdf]

Supplementary Information for

# Flow-induced periodic chiral structures in an achiral nematic liquid crystal

Qing Zhang[1,a,*], Weiqiang Wang[2,a], Shuang Zhou[3], Rui Zhang[2,*], Irmgard Bischofberger[1,*]

[1]Department of Mechanical Engineering, Massachusetts Institute of Technology, Cambridge, MA 02139, USA

[2]Department of Physics, Hong Kong University of Science and Technology, Hong Kong, China

[3]Department of Physics, University of Massachusetts Amherst, Amherst, MA 01003, USA

*e-mail: zqing@mit.edu, ruizhang@ust.hk, irmgard@mit.edu

**The PDF file includes:**

Supplementary Information

Figs. S1 to S9

**Other Supplementary Information for this manuscript includes the following:**

Supplementary Video 1

## Director field of stripe pattern

Using a polarising optical microscope, we identify the director field at the dark stripes by rotating the crossed polariser and analyser by 45°. The observation that the dark stripes remain dark indicates that the director is homeotropically aligned in this region, as shown in Fig. S1(a). The dark stripes correspond to region I in Fig. 1(c). To determine the director field between two dark stripes (region II in Fig. 1(c)), we add a static full-wave-plate optical compensator (560 nm) with the slow axis, $\vec{\lambda}_{\rm g}$, oriented at 45° to the crossed polarisers and in the direction perpendicular to the $x$-direction. We then compare the birefringence colours with the Michel-Lévy colour chart[1]. In experiments performed without the full-wave-plate optical compensator, the retardance in between two dark stripes is approximately 150 nm (light grey colour), as shown in Fig. S1(a). After the compensator is inserted, a change in colour, representing a change in the retardance, gives information about the azimuthal angle of the director field: an increase of the retardance by 560 nm indicates that the director is perpendicular to $\vec{\lambda}_{\rm g}$; a decrease of the retardance by 560 nm indicates that the director is parallel to $\vec{\lambda}_{\rm g}$. We measure a retardance of approximately 410 nm = |560-150| nm between two dark stripes (change in birefringence colour from light grey (150 nm) to orange (410 nm)), as shown in Fig. S1(a). The director field of disodium cromoglycate (DSCG) solutions is thus almost parallel to $\vec{\lambda}_{\rm g}$, *i.e.*, almost perpendicular to the flow direction. To more precisely quantify the azimuthal angle in between the low retardance regions (region I) averaged in the gap thickness direction, we use a PolScope and find that it is alternatively $\approx 62°$ and $\approx 115°$, as shown in Fig. S1(b, c). Overall, the director thus rotates from aligning in the $y$-direction to aligning in the $z$-direction, which indicates a twist deformation along the flow direction.

## Azimuthal angle of nematic DSCG solutions in the regions of uniform director field

To investigate the azimuthal angle of the director in the regions where the director field is uniform, we simulate the director field using the hybrid lattice Boltzmann method[2,3]. The surface anchoring condition is planar, so that the azimuthal angle is 0°. For a low pressure gradient, $G$, the azimuthal angle in the centre of the microfluidic cell is 0° consistent with the surface anchoring condition, as shown in Fig. S2. For a high pressure gradient, the azimuthal angle is $\approx 90°$ corresponding to a log-rolling state[4]. For intermediate pressure gradients, the azimuthal angle adopts a rotated state that is in agreement with the experimental observations.

**Nematodynamic equation describing the evolution of the director field at the wall of the microfluidic cell**

The nematodynamic equation describing the evolution of the director at the two walls of the microfluidic cell reads[5]

$$k\left(\frac{d\mathbf{n}}{dt}\right)_{surface} = \hat{v}\cdot\frac{\partial f_{bulk}}{\partial\nabla\mathbf{n}} + \frac{\partial f_{surface}}{\partial\mathbf{n}}, \tag{S1}$$

where $\mathbf{n} = (\sin\theta\cos\varphi, \sin\theta\sin\varphi, \cos\theta)$ is the director field with $\theta$ the polar angle and $\varphi$ the azimuthal angle, $k$ is related to the rotational viscosity of the lyotropic chromonic liquid crystals (LCLC) solution, and $\hat{v}$ is the surface normal. The subscript '$surface$' denotes the director at the surface of the walls. $f_{bulk} = \frac{1}{2}K_1(\nabla\cdot\mathbf{n})^2 + \frac{1}{2}K_2(\mathbf{n}\cdot\nabla\times\mathbf{n})^2 + \frac{1}{2}K_3(\mathbf{n}\times(\nabla\times\mathbf{n}))^2$ is the bulk term in the Oseen-Frank elastic free energy density and $f_{surface} = -\frac{1}{2}K_{24}\nabla\cdot((\mathbf{n}(\nabla\cdot\mathbf{n})) + \mathbf{n}\times(\nabla\times\mathbf{n}))$ is the surface term.

For the flowing nematic DSCG solutions, we have $\hat{v} = (0,0,1)$ at the top wall of the microfluidic cell. We assume that $d\varphi/dx = 0$ and $d\theta/dx = 0$ in steady state flow. We consider the vicinity of the symmetry axis in the $xy$-plane of the biaxial-splay and double-splay regions, where the director is predominantly oriented in the shear plane so that $\varphi \approx 0°$. The contribution of the bulk term, $f_{bulk}$, to the evolution of the director at the walls of the microfluidic cell in Eq. S1 then simplifies to

$$\hat{v}\cdot\frac{\partial f_{bulk}}{\partial\nabla\mathbf{n}} \approx -\bar{K}\left(\frac{\partial\theta}{\partial z}\right)_{surface}, \tag{S2}$$

where $\bar{K} = (K_1 + K_3)/2$. The contribution of the surface term, $f_{surface}$, to the evolution of the director at the walls of the microfluidic cell can be expressed as

$$\frac{\partial f_{surface}}{\partial\mathbf{n}} \approx \frac{1}{2}K_{24}\left(\cos^2\theta - \sin^2\theta\right)\frac{\partial\varphi}{\partial y}. \tag{S3}$$

Substituting Eqs. S2 and S3 into Eq. S1 yields

$$k\left(\frac{d\theta}{dt}\right)_{surface} = -\bar{K}\left(\frac{\partial\theta}{\partial z}\right)_{surface} + \frac{1}{2}K_{24}\left(\cos^2\theta - \sin^2\theta\right)\frac{\partial\varphi}{\partial y}. \tag{S4}$$

We nondimensionalise Eq. S4 using $y^* = y/w$, $z^* = z/b$, and $t^* = t/\tau$, where $b$ is the gap thickness, $w \approx b/2$ is the characteristic width of the double-splay and biaxial-splay configurations, and $\tau$ is the characteristic relaxation time of the director:

$$\frac{kw}{\bar{K}\tau}\left(\frac{d\theta}{dt^*}\right)_{surface} = -\frac{w}{b}\left(\frac{\partial\theta}{\partial z^*}\right)_{surface} + \frac{1}{2}\frac{K_{24}}{\bar{K}}\left(\cos^2\theta - \sin^2\theta\right)\frac{\partial\varphi}{\partial y^*}. \tag{S5}$$

For the double-splay configuration, $\frac{\partial\varphi}{\partial y^*} > 0$ and the term scales as $\frac{\partial\varphi}{\partial y^*} \propto 1$. For the biaxial-splay configuration, $\frac{\partial\varphi}{\partial y^*} < 0$ and the term scales as $\frac{\partial\varphi}{\partial y^*} \propto -1$.

To solve Eq. S5 and to analyse the time evolution of the director at the walls of the microfluidic cell, we need to know the distribution of $\theta$ in the $z$-direction that is obtained by solving the one-dimensional nematodynamic equation in the bulk[6]:

$$\gamma_1 \left(\frac{d\theta}{dt}\right)_{bulk} = \bar{K}\frac{\partial^2\theta}{\partial z^2} - \dot{\gamma}\left(\alpha_2 \cos^2\theta - \alpha_3 \sin^2\theta\right), \tag{S6}$$

where $\gamma_1$ is the rotational viscosity and $\alpha_2$ and $\alpha_3$ are the Leslie viscosity coefficients. To solve Eq. S6 for a steady state flow, where $\gamma_1\left(\frac{d\theta}{dt}\right)_{bulk} = 0$, we need to determine the boundary condition that is given by the polar angle at the two walls of the microfluidic cell, $\theta_b$.

To find a realistic $\theta_b$ for our weak surface anchoring condition in shear flow, we firstly assume different values for $\theta_b$ ranging from 45° to 90°. From Eq. S6 in steady state flow where $\left(\frac{d\theta}{dt}\right)_{bulk} = 0$, each of these presumed $\theta_b$ yields a distribution of $\theta$ in the gap thickness direction, as shown in Fig. S3(a), which gives the gradient of $\theta$ in the $z$-direction at the two walls of the microfluidic cell, $\left(\frac{\partial\theta}{\partial z^*}\right)_{surface}$. Inserting the values of $\left(\frac{\partial\theta}{\partial z^*}\right)_{surface}$ into Eq. S5 yields the dependence of $\frac{kw}{K\tau}\left(\frac{d\theta}{dt^*}\right)_{surface}$ on $\theta$ at the walls of the microfluidic cell for the biaxial-splay configuration, as shown in Fig. S3(a). At the walls, where the director is stationary because of the no-slip boundary condition, $\frac{kw}{K\tau}\left(\frac{d\theta}{dt^*}\right)_{surface} = 0$, and we get the stationary angle at the walls of the microfluidic cell denoted as $\theta_s$. If $\theta_b > \theta_s$, $\frac{kw}{K\tau}\left(\frac{d\theta}{dt^*}\right)_{surface}$ is positive, which means that $\theta$ would increase with time and reach 90°, *i.e.*, a planar alignment. By contrast, if $\theta_b < \theta_s$, $\frac{kw}{K\tau}\left(\frac{d\theta}{dt^*}\right)_{surface}$ is negative and $\theta$ decreases to 0°, a homeotropic alignment.

We plot the value of $\frac{kw}{K\tau}\left(\frac{d\theta}{dt^*}\right)_{surface}$ for the different presumed $\theta_b$ in Fig. S3(c). This plot elucidates whether the director for different $\theta_b$ in the biaxial-splay configuration will evolve toward a homeotropically or a planarly aligned state, and identifies a critical angle, $\theta_c$, for which $\frac{kw}{K\tau}\left(\frac{d\theta}{dt^*}\right)_{surface} = 0$. For $\theta_b < \theta_c$, the director evolves to a homeotropic alignment.

Within the range of Ericksen number $\mathrm{Er_{av}} = -\alpha_2\dot{\gamma}b^2/\bar{K} = 25$–50 where the stripe patterns form (corresponding to $\mathrm{Er} = \eta_{eff}Vb/K_3 = 0.65$–1.25 using the definition employed in the main manuscript), the critical angle, $\theta_c$, increases with increasing $\mathrm{Er_{av}}$, as shown in Fig. S3(d). How strongly $\theta_c$ deviates from the imposed surface anchoring condition, $\theta_{b,imposed} = 90°$, reflects the surface anchoring strength. At lower $\mathrm{Er_{av}}$, a weaker surface anchoring strength is required to induce homeotropic alignment.

We estimate the surface anchoring strength, $W$, for different $\theta_c$ in the range of $\mathrm{Er_{av}} = 25$–50. $W$ is evaluated

by determining the extrapolation length, $L_0$, which is the length obtained by extrapolating $\theta$ from $\theta_c$ at the walls of the microfluidic cell to 90°, as shown in Fig. S4(a). The ratio between the surface distortion energy and the bulk distortion energy equals the ratio between $L_0$ and the gap thickness $b$[7]:

$$\frac{F_{surface}}{F_{bulk}} = \frac{L_0}{b}, \tag{S7}$$

where $F_{surface} = \frac{1}{2}W\theta_b^2$ and $F_{bulk} = \frac{1}{2}\int_0^b \bar{K}\left(\frac{\partial\theta}{\partial y}\right)^2 dy$. This yields an expression for the surface anchoring strength:

$$W = \frac{L_0}{b\theta_b^2}\int_0^b \bar{K}\left(\frac{\partial\theta}{\partial y}\right)^2 dy \approx \frac{L_0\bar{K}}{b^2}. \tag{S8}$$

For $\theta_c$ obtained in the regime of $\mathrm{Er}_{\mathrm{av}}$ = 25–50, $W$ is on the order of $10^{-6}$ $\mathrm{J/m^2}$, as shown in Fig. S4(a). Experimentally, the polar surface anchoring strength of DSCG solutions on rubbed glass surfaces has not yet been determined. To ensure our estimated values are within a realistic range, we draw an analogy with research on Bacillus subtilis in a lyotropic chromonic liquid crystal (LCLC) solution in a cell with homeotropic alignment[8]. In this study, the initial alignment of the bacteria is parallel to the director field, where the director on the bacteria's surface is planarly aligned and parallel to the long axis of the bacteria. As the bacteria rotate and deviate from this initial state, the director field around the bacteria exhibits a splay deformation, extending from the bacteria surface to the far field. This scenario involves a competition between the polar anchoring strength on the bacteria surface and the torque exerted by the bacteria flagella motors. The study employs numerical simulations to estimate the polar anchoring strength on the bacteria surface allowing the bacteria to be perpendicular to the LCLC solutions director field, and reports a value of $10^{-6}$ $\mathrm{J/m^2}$. This value is in agreement with our values obtained by assuming $\theta_b = \theta_c$ as the boundary condition for solving Eq. S6. This provides justification for our numerical procedure. However, it is important to note that the value of surface anchoring strength derived in the study of Bacillus subtilis may not exactly represent the polar anchoring strength on rubbed glass surfaces, and we acknowledge that direct measurements of the polar anchoring strength on rubbed glass surfaces will provide important further insight.

**Elastic energy of different director configurations**

In the experiments, we have identified that the director field of DSCG solutions can adopt three director configurations: biaxial-splay, double-splay and double-twist configurations. We describe these configurations in cylindrical coordinates. For the biaxial-splay configuration, the director field is $\mathbf{n} = (\sin\theta\cos\alpha, \sin\theta\sin\alpha, \cos\theta)$, where $\theta(\rho)$ is the polar angle and $\alpha = -2\phi$ is the azimuthal angle between the director and the $\rho$-axis, as shown

in Fig. S5. Substituting the director field of the biaxial-splay configuration into the Oseen-Frank elastic free energy density[6],

$$f = 1/2[K_1(\nabla\cdot\mathbf{n})^2 + K_2(\mathbf{n}\cdot\nabla\times\mathbf{n})^2 + K_3(\mathbf{n}\times\nabla\times\mathbf{n})^2 - K_{24}\nabla\cdot(\mathbf{n}(\nabla\cdot\mathbf{n}) + \mathbf{n}\times(\nabla\times\mathbf{n}))], \quad \text{(S9)}$$

yields

$$\begin{aligned} f_{biaxial-splay} = &\frac{1}{2}K_1\cos^2\alpha\left(\cos\theta\frac{\partial\theta}{\partial\rho} - \frac{\sin\theta}{\rho}\right)^2 + \frac{1}{2}K_2\sin^2\alpha\left(\frac{\partial\theta}{\partial\rho} - \frac{\sin\theta\cos\theta}{\rho}\right)^2 \\ &+ \frac{1}{2}K_3\sin^2\theta\left(\frac{\sin^2\theta\sin^2\alpha}{\rho^2} + \cos^2\alpha\left(\frac{\partial\theta}{\partial\rho}\right)^2\right) + K_{24}\left(\frac{\sin\theta\cos\theta}{\rho}\frac{\partial\theta}{\partial\rho}\right). \quad \text{(S10)} \end{aligned}$$

For the double-splay configuration, the director field is $\mathbf{n} = (\sin\theta, 0, \cos\theta)$. The Oseen-Frank elastic free energy density is

$$f_{double-splay} = \frac{1}{2}K_1\left(\cos\theta\left(\frac{\partial\theta}{\partial\rho}\right) + \frac{\sin\theta}{\rho}\right)^2 + \frac{1}{2}K_3\sin^2\theta\left(\frac{\partial\theta}{\partial\rho}\right)^2 - K_{24}\left(\frac{\sin\theta\cos\theta}{\rho}\frac{\partial\theta}{\partial\rho}\right). \quad \text{(S11)}$$

For the double-twist configuration, the director field is $\mathbf{n} = (0, \sin\theta, \cos\theta)$. The Oseen-Frank elastic free energy density is

$$f_{double-twist} = \frac{1}{2}K_2\left(\frac{\sin\theta\cos\theta}{\rho} + \left(\frac{\partial\theta}{\partial\rho}\right)\right)^2 + \frac{1}{2}K_3\left(\frac{\sin^4\theta}{\rho^2}\right) - K_{24}\left(\frac{\sin\theta\cos\theta}{\rho}\frac{\partial\theta}{\partial\rho}\right). \quad \text{(S12)}$$

A comparison of Eqs. S10, S11 and S12 reveals that the saddle-splay elasticity term, $K_{24}\left(\frac{\sin\theta\cos\theta}{\rho}\frac{\partial\theta}{\partial\rho}\right)$, is negative for the double-twist and double-splay configurations, but positive for the biaxial-splay configuration. The saddle-splay elasticity thus lowers the energy for the double-splay and double-twist configurations, but increases the energy for the biaxial-splay configuration. Given that $K_{24} \approx K_3 \approx 3K_1 \approx 30K_2$[9, 10], $f_{biaxial-splay}$ is larger than $f_{double-splay}$ and $f_{double-twist}$, as shown in Fig. S6. The double-twist configuration is the energetically most favorable configuration and is selected.

**Elastic powers evaluated in simulations**

From the hybrid lattice Boltzmann simulations, we calculate the temporal evolution of the elastic powers of splay, twist, bend and saddle-splay deformations, denoted as $P_{splay} = \int_\Lambda (\nabla\cdot\mathbf{n})^2 d\Lambda$, $P_{twist} = \int_\Lambda (\mathbf{n}\cdot(\nabla\times\mathbf{n}))^2 d\Lambda$, $P_{bend} = \int_\Lambda (\mathbf{n}\times(\nabla\times\mathbf{n}))^2 d\Lambda$, and $P_{saddle-splay} = \int_\Lambda -\nabla\cdot(\mathbf{n}(\nabla\cdot\mathbf{n}) + \mathbf{n}\times(\nabla\times\mathbf{n}))d\Lambda$, where $\Lambda$ is a control volume (Fig. S7). Prior to the occurrence of mirror symmetry breaking, $P_{twist}$ and $P_{saddle-splay}$ are

comparable. As the double-twist configuration emerges, $P_{twist}$ increases and becomes an order of magnitude larger than $P_{saddle-splay}$. This confirms that the twist mode is energetically favorable.

**Handedness of the chiral periodic double-twist configuration**

To determine the selection of handedness of the periodic double-twist configuration, we solve the Beris-Edwards equation[2] using the hybrid lattice Boltzmann method on a D3Q19 lattice[11]. We confine the liquid crystal to a box with dimensions $[L_x,\ L_y,\ L_z]$ = [151, 151, 51] and impose finite planar surface anchoring on both walls. We use periodic boundary conditions in the $x$- and $y$-directions, and we set $A_0 = 0.05$, $L_1 = 0.05$, $L_2$ = 0, $L_3$ = 0.1624, and $L_4 = 0.0667$, corresponding to $K_1 = 3K_2 = \frac{1}{3}K_3 = K_{24}$, and flow-tumbling characteristics ($\xi = 0.6$) to describe nematic DSCG solutions[9, 12]. Upon application of a pressure-driven flow along the $x$-direction, the periodic double-twist configuration emerges robustly, irrespective of whether we start the simulations from a random initial condition or from a uniform initial condition. We find that the periodic double-twist configuration has the same handedness along the $x$- and $z$-directions, as shown in the snapshot in Fig. S8 and in Table S1). The same handedness is selected in both twist directions as it leads to a smooth variation of the twisting and avoids additional energetically costly splay deformations that would be needed for opposite-handedness of the twist. Left- and right-handedness are stochastically equal. In 21 independent simulations, we find 10 left-handed and 11 right-handed configurations, as reported in Table S1.

**Table S1.** Handedness of the periodic double-twist configuration, obtained in 21 independent simulations.

| | left-handed twist in $x$-direction | right-handed twist in $x$-direction |
|---|---|---|
| left-handed twist in $z$-direction | 10 | 0 |
| right-handed twist in $z$-direction | 0 | 11 |

**Derivation of the characteristic period of the stripes**

Given that the twist Frank elastic constant is much smaller than the splay and bend Frank elastic constants, $K_2 << K_{1,3}$, we neglect the contribution of twist deformation to the total energy of the periodic double-twist structure and consider the region close to wall of the microfluidic cell that is dominated by bend and splay deformations. The director field is $\mathbf{n} = (\sin\theta, 0, \cos\theta)$, where $\theta$ is the polar angle that is assumed to be only a

function of $x$ in this region. The nematodynamic equation in a steady state flow is expressed as[6]

$$(K_1 - K_3)\cos\theta\sin\theta\left(\frac{\partial\theta}{\partial x}\right)^2 + \left(K_3\sin^2\theta + K_1\cos^2\theta\right)\frac{\partial^2\theta}{\partial x^2} = \left(\alpha_2\cos^2\theta - \alpha_3\sin^2\theta\right)\dot{\gamma}_{xz}, \tag{S13}$$

where $\dot{\gamma}_{xz}$ is the shear rate in the $xz$-plane. For simplicity, we assume $\theta \approx 90°$ in the region close to the walls of the microfluidic cell, so that $\alpha_2\cos^2\theta - \alpha_3\sin^2\theta \approx -\alpha_3$, $(K_1 - K_3)\cos\theta\sin\theta \approx 0$, and $K_3\sin^2\theta + K_1\cos^2\theta \approx K_3$. Eq. S13 then yields

$$\alpha_3\dot{\gamma}_{xz} = -K_3\frac{\partial^2\theta}{\partial x^2}. \tag{S14}$$

The characteristic length scale of the bend deformation in the $x$-direction scales as half of the period of the stripes, $p_c/2$, and the shear rate scales as $\dot{\gamma}_{xz} \propto V/b$, where $V$ is the velocity of the stripes. The elastic term can then be expressed as $K_3/(p_c/2)^2$ and the viscous term as $\alpha_3 V/b$. The balance between the elastic term and the viscous term, $K_3/(p_c/2)^2 \propto \alpha_3 V/b$, yields an expression for the period of the stripes: $p_c \propto 2\sqrt{K_3 b/\alpha_3 V}$. This expression is in agreement with the experimentally observed dependence of the period of the stripes on $V$ and $b$.

**Effective viscosity of DSCG solutions**

To determine the effective viscosity of a 13 wt% DSCG solution, $\eta_{eff}$, in the range of shear rates corresponding to those occurring in the stripe pattern experiments, we use a stress-controlled rheometer (AR-G2, TA Instruments) equipped with a cone-plate geometry. We report $\eta_{eff}$ as the average of the viscosity $\eta_{\mathrm{DSCG}}$ measured in the range of shear rates $\dot{\gamma}_{\mathrm{r}} = \dot{\gamma}_{xz}$ indicated by the grey region in Fig. S9.

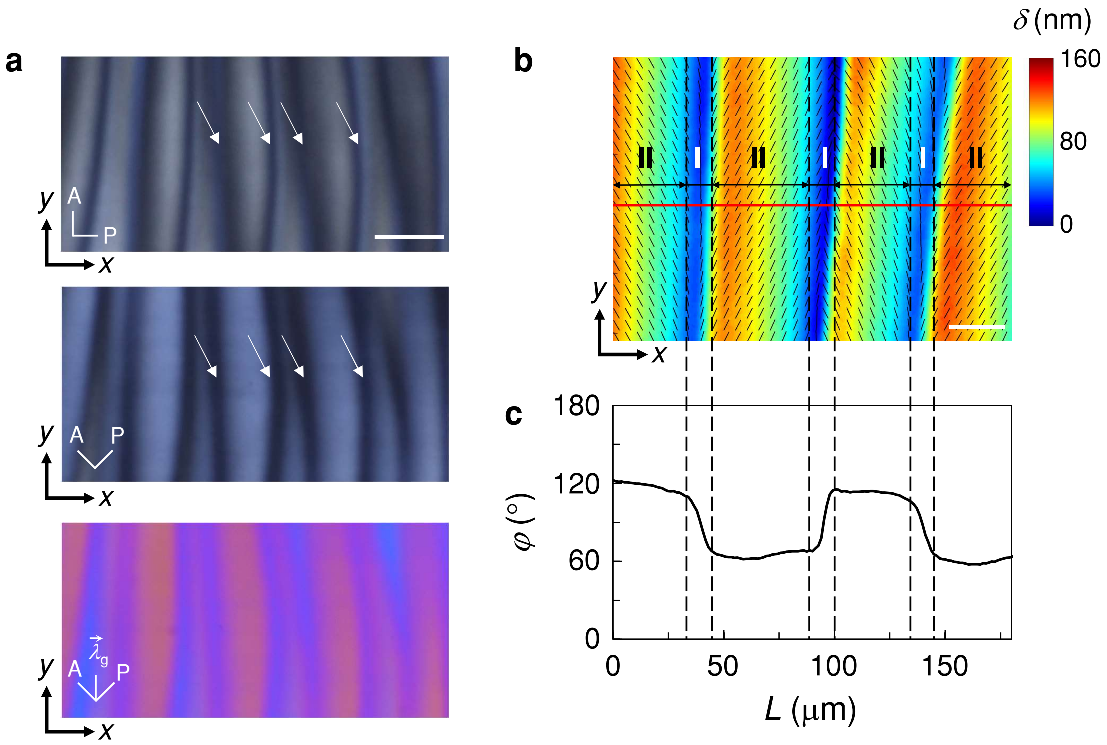


**Fig. S1. Director field of stripe patterns.** (**a**) The dark stripes (indicated by arrows) remain dark upon a rotation of the crossed polariser, P, and analyser, A, by 45° (top and middle panels). Upon addition of a full-wave-plate optical compensator with its slow axis, $|\vec{\lambda}_g|$ = 560 nm, perpendicular to the flow direction, regions in between the dark stripes appear orange indicating a director alignment in the $y$-direction (lower panel). The scale bar is 100 $\mu$m. (**b**) Retardance map of the stripe pattern. The colour represents the optical retardance, $\delta$, and the direction of the black rods denotes the orientation of the director averaged in the gap thickness direction projected onto the $xy$-plane for a cell with gap thickness $b = 20$ $\mu$m. Region I denotes the dark stripes observed under crossed polariser and analyser, region II denotes the regions in between the dark stripes. The scale bar is 25 $\mu$m. (**c**) The gap-averaged azimuthal angle, $\varphi$, measured along the red line indicated in (b) alternates between $\approx 62°$ and $\approx 115°$.

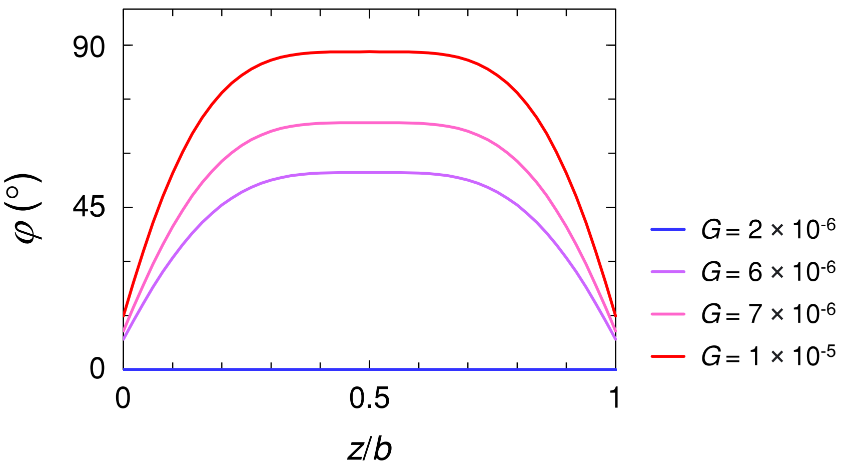


**Fig. S2. Azimuthal angles for different pressure gradients.** With increasing pressure gradient, $G$, the azimuthal angle, $\varphi$, in the centre region of the microfluidic cell increases from 0° (direction of surface anchoring) to $\approx 90°$. At intermediate $G$, the director is in a rotated state, in agreement with the experimental observations.

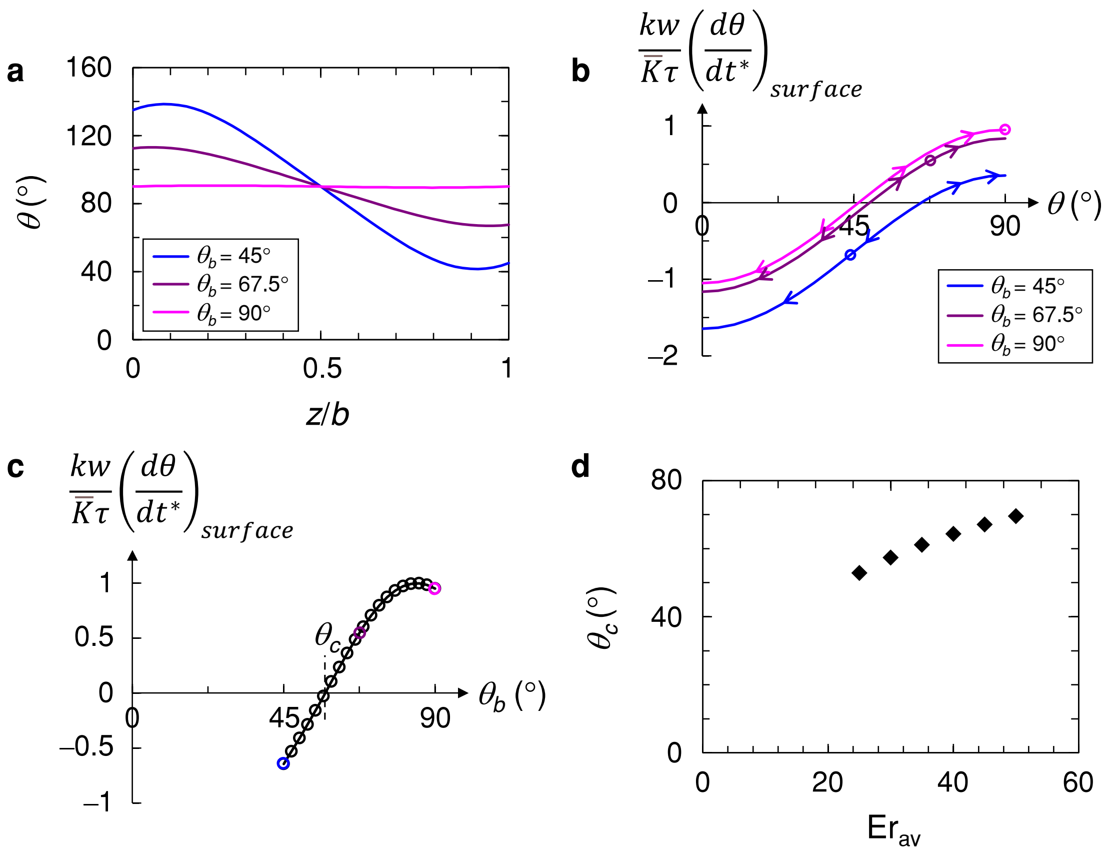


**Fig. S3. Determination of the polar angle at the walls of the microfluidic cell and at the splay wall formed by a biaxial-splay configuration.** (**a**) The polar angle, $\theta$, across the gap thickness depends on the imposed polar angle at the walls of the microfluidic cell, $\theta_b$. (**b**) For $\theta_b = 45°$, the polar angle at the walls of the microfluidic cell evolves towards 0°. For $\theta_b = 67.5°$ and $\theta_b = 90°$, the polar angle at the walls of the microfluidic cell evolves towards 90°. The open circles represent $\theta_b$. (**c**) The value $\frac{kw}{\bar{K}\tau}\left(\frac{d\theta}{dt^*}\right)_{surface}$ for different presumed $\theta_b$. The condition $\frac{kw}{\bar{K}\tau}\left(\frac{d\theta}{dt^*}\right)_{surface} = 0$ provides the critical polar angle, $\theta_c$. For $\theta_b > \theta_c$, the director evolves towards 90° (planar alignment). For $\theta_b < \theta_c$, the director evolves towards 0° (homeotropic alignment). In (a-c), $\mathrm{Er}_{\mathrm{av}}$ = 30. (**d**) The critical polar angle $\theta_c$ depends on $\mathrm{Er}_{\mathrm{av}}$.

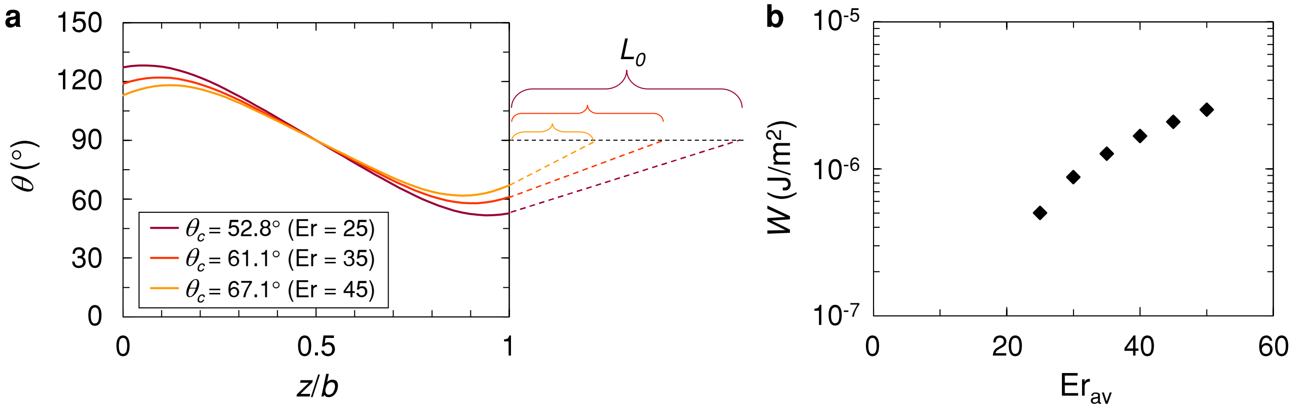


**Fig. S4. Evaluation of the surface anchoring strength.** (**a**) Polar angle, $\theta$, across the gap thickness, calculated for different critical polar angles, $\theta_c$. The extrapolation length, $L_0$, is obtained by extrapolating the data from the wall of the microfluidic cell to the location where $\theta$ reaches $90°$. (**b**) The surface anchoring strength, $W$, versus $\mathrm{Er}_{\mathrm{av}}$.

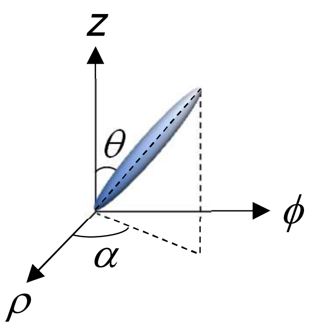


**Fig. S5. Director orientation in cylindrical coordinates.** $\alpha$ is the azimuthal angle and $\theta$ is the polar angle.

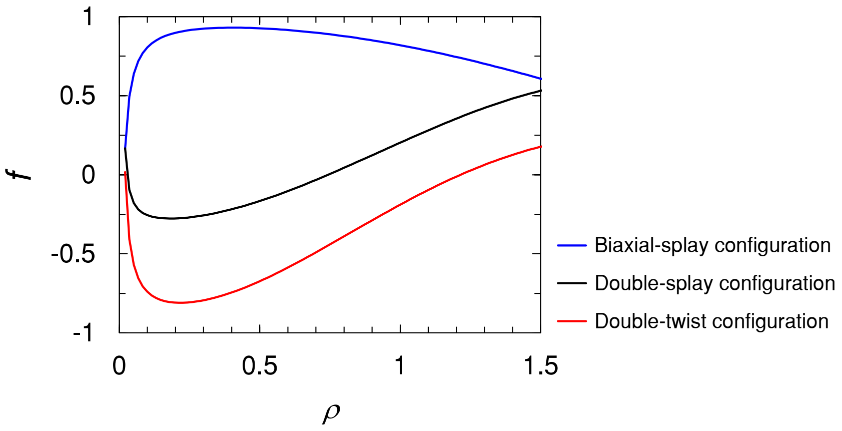


**Fig. S6. Oseen-Frank elastic free energy densities of the three director configurations.** The Oseen-Frank elastic free energy density $f$ of the double-twist configuration is lower than those of the biaxial-splay and double-splay configurations.

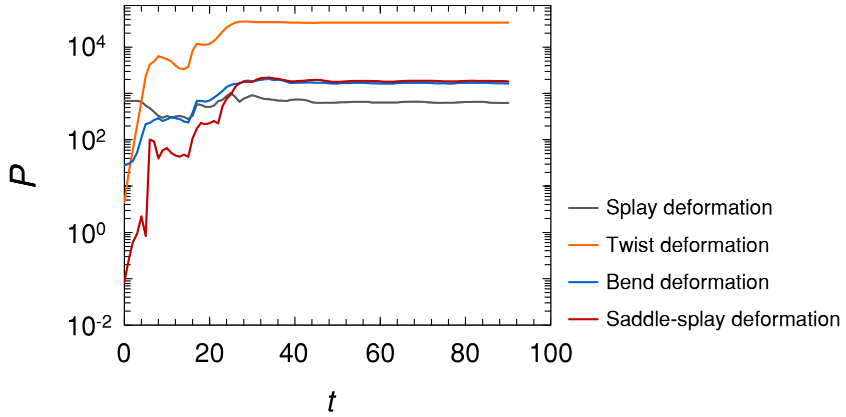


**Fig. S7. Elastic powers of different deformation modes in simulations.** Temporal evolution of the elastic powers $P$ of splay, twist, bend and saddle-splay deformations upon the onset of flow.

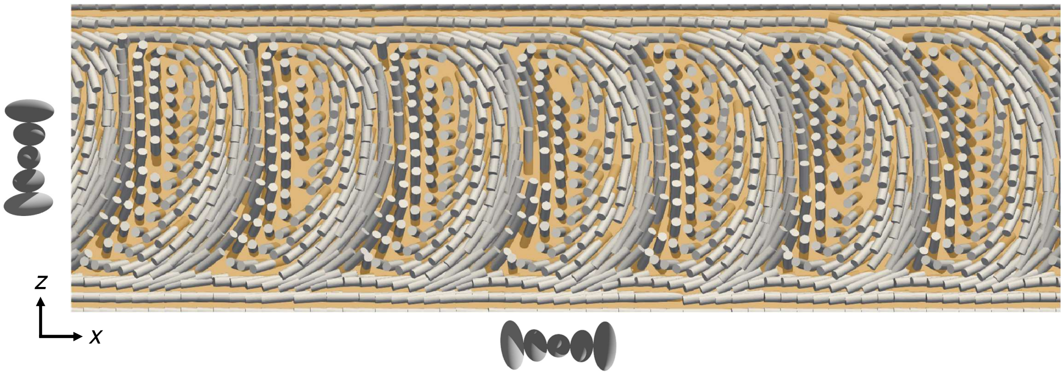


**Fig. S8. Handedness of the flow-induced periodic double-twist configuration, assessed in simulations.** The director field of the periodic double-twist configuration exhibits the same handedness in both the $x$- and $z$-directions (right-handed chirality in this specific example).

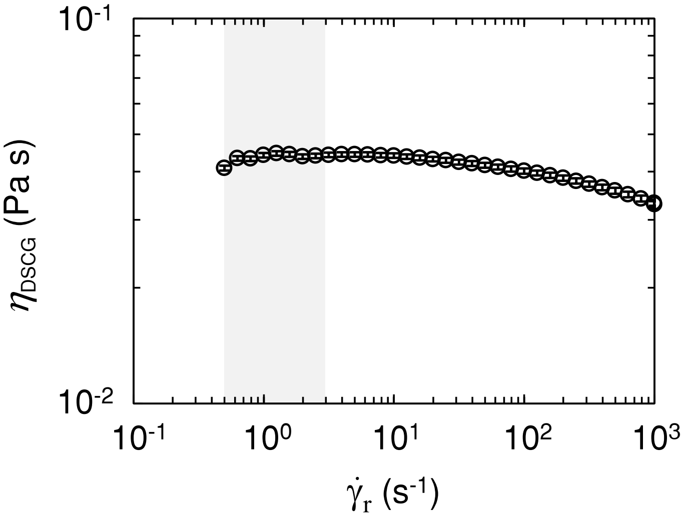


**Fig. S9. Effective viscosity of a 13 wt% DSCG solution versus shear rate measured in rheology.** The range of shear rates relevant for the stripe pattern experiments is highlighted in grey.

# References


1. Sørensen, B. E. A revised Michel-Lévy interference colour chart based on first-principles calculations. *Eur. J. Mineral.* **25,** 5–10 (2013).
2. Zhang, R., Roberts, T., Aranson, I. S. & De Pablo, J. J. Lattice Boltzmann simulation of asymmetric flow in nematic liquid crystals with finite anchoring. *J. Chem. Phys.* **144,** 084905 (2016).
3. Denniston, C., Marenduzzo, D., Orlandini, E. & Yeomans, J. Lattice Boltzmann algorithm for three–dimensional liquid–crystal hydrodynamics. *Philos. Trans. Royal Soc. A* **362,** 1745–1754 (2004).
4. Baza, H. *et al.* Shear-induced polydomain structures of nematic lyotropic chromonic liquid crystal disodium cromoglycate. *Soft Matter* **16,** 8565–8576 (2020).
5. Beris, A. N. & Edwards, B. J. *Thermodynamics of flowing systems: with internal microstructure* (Oxford University Press, 1994).
6. Kleman, M. & Lavrentovich, O. D. *Soft matter physics: an introduction* (Springer Science & Business Media, 2007).
7. De Gennes, P.-G. & Prost, J. *The physics of liquid crystals* (Oxford University Press, 1993).
8. Zhou, S. *et al.* Dynamic states of swimming bacteria in a nematic liquid crystal cell with homeotropic alignment. *New J. Phys.* **19,** 055006 (2017).
9. Zhang, Q. *et al.* Structures and topological defects in pressure-driven lyotropic chromonic liquid crystals. *Proc. Natl. Acad. Sci. U. S. A.* **118** (2021).
10. Chang, R. *Chiral configurations from achiral lyotropic chromonic liquid crystals under confinements* PhD thesis (Georgia Institute of Technology, 2018).
11. Krüger, T. *et al. The lattice Boltzmann method* (Springer, 2017).
12. Wang, W. & Zhang, R. Interplay of active stress and driven flow in self-assembled, tumbling active nematics. *Crystals* **11,** 1071 (2021).
