# Supplementary material for: Flow-induced periodic chiral structures in an achiral nematic liquid crystal
Source: Nat Commun. 2024 Jan 8;15:7. doi: 10.1038/s41467-023-43978-6 (PMC10774319; doi:10.1038/s41467-023-43978-6)
Supplement: Supplementary file 3 — Description of Additional Supplementary Files [file 41467_2023_43978_MOESM3_ESM.docx]

**Description of Additional Supplementary Files**

**File Name: Supplementary Video 1
Description:** Stripe pattern emerging in nematic DSCG solutions at a volumetric flow rate *q* = 0.25 μl/min. P denotes the polariser and A denotes the analyser. The video is accelerated five times.
